# Astrocyte TrkB.T1 Deficiency Disrupts Glutamatergic Synaptogenesis and Astrocyte–Synapse Interactions

**DOI:** 10.1523/JNEUROSCI.2002-24.2026

**Published:** 2026-07-10

**Authors:** Beatriz T. C. Pinkston, Jack L. Browning, Amelie P. Larson, Michelle L. Olsen

**Affiliations:** School of Neuroscience, Virginia Polytechnic and State University, Blacksburg, Virginia 24061

**Keywords:** astrocyte, BDNF, synapse development, TrkB

## Abstract

Perisynaptic astrocyte processes (PAPs) contact pre- and postsynaptic elements to provide structural and functional support to synapses. Accumulating research demonstrates that the contact of synapses by PAPs is critical for synapse formation, stabilization, and plasticity. The specific signaling pathways that govern these astrocyte–synapse interactions, however, remain to be elucidated. Herein, we demonstrate the role of the astrocyte TrkB.T1 receptor, a truncated isoform of the canonical receptor for brain-derived neurotrophic factor (BDNF), in modulating astrocyte–synapse interactions and excitatory synapse development. Neuron–astrocyte coculture studies revealed that loss of astrocyte TrkB.T1 disrupts the formation of PAPs. To elucidate the role of TrkB.T1 in synapse development, we conditionally deleted TrkB.T1 in astrocytes in mice of either sex. Synaptosome preparations were employed to probe for TrkB.T1 localization at the PAP, and confocal three-dimensional microscopy revealed a significant reduction in synapse density and astrocyte–synapse interactions across development in the absence of astrocytic TrkB.T1. Furthermore, conditional knock-out of astrocyte TrkB.T1 alters motor learning and experience-dependent astrocyte–synapse interactions. These findings suggest that BDNF/TrkB.T1 signaling in astrocytes is critical for normal excitatory synapse formation in the cortex and that astrocyte TrkB.T1 serves a requisite role in astrocyte synapse interactions. Overall, this work provides new insights into the molecular mechanisms of astrocyte-mediated synaptogenesis.

## Significance Statement

The role of astrocytes at the synapse is critical, and understanding the mechanisms that guide their interactions is essential for unraveling brain development. By demonstrating that the astrocyte TrkB.T1 receptor is critical for the formation of perisynaptic astrocyte processes and their interactions with synapses, this study provides new insights into how astrocytes support synapse development. These findings enhance our understanding of the molecular mechanisms underlying brain development and plasticity. By elucidating the importance of brain-derived neurotrophic factor/TrkB.T1 signaling in astrocytes, this work bridges a significant gap in our knowledge of astrocyte–synapse interactions and may lead to novel therapeutic strategies targeting astrocyte function in neurological conditions.

## Introduction

Brain-derived neurotrophic factor (BDNF) and its receptor tropomyosin receptor kinase B (TrkB) constitute a powerful molecular partnership that serve to govern neuronal survival and maturation as well as synapse formation and plasticity, thereby shaping the intricate neuronal circuits that underlie mammalian behavior. While the role of BDNF/TrkB signaling in the development and maintenance of synapses has been extensively studied, we recently unveiled an unexpected player in this molecular pathway—the astrocyte.

We have demonstrated that astrocytes predominantly and nearly exclusively express a truncated isoform of the TrkB receptor, TrkB.T1 (Holt et al., 2019a). Furthermore, evaluation of published sequencing data indicates that TrkB.T1 is the primary isoform identified in the cortex of both mice (Zhang et al., 2014; Chai et al., 2017) and humans (Zhang et al., 2016 ; Kelley et al., 2018), and astrocytes across species are the highest expressers of TrkB (Wei et al., 2024) suggesting that astrocytes are a major recipient of BDNF in the cortex. Astrocytes are a major glial cell type that has emerged as critical regulators in synapse development and stabilization. These functions are facilitated by their morphological complexity, which is best described as a dynamic spongiform shape, made up by thousands of fine, leaflet-like processes, often referred to as perisynaptic astrocyte processes (PAPs) that infiltrate the neuropil and allow a singular mammalian astrocyte to contact up to 100,000 synapses at a time (Bushong et al., 2002). From a functional standpoint, PAPs isolate synapses by forming a physical barrier that facilitates the maintenance of both ions (Olsen and Sontheimer, 2008; Nwaobi et al., 2016 ; Armbruster et al., 2022) and neurotransmitters (Anderson and Swanson, 2000) in the synaptic space. PAPs are enriched with numerous transporters, receptors, and ion channels, such as the glutamate transporters GLT1 and glutamate aspartate transporter (GLAST; Perego et al., 2000), the GABA transporter GAT3 (Minelli et al., 1996), and the inwardly rectifying potassium channel-4.1 Kir4.1 (Olsen and Sontheimer, 2008; Olsen, 2012). Together these proteins work to remove neuronally derived neurotransmitter and K^+^ ions from the synaptic cleft following neuronal activity (Yang et al., 2009; Armbruster et al., 2022). Astrocytes are also key modulators in the development and maturation of synapses through their expression of critical cell adhesion molecules and secretion of synaptogeneic cues (Chung et al., 2015). Astrocytes have been increasingly implicated in the control of behavior (Kofuji and Araque, 2021; Lyon and Allen, 2021; Barnett et al., 2023); however, the molecular mechanisms that direct astrocytic processes to synapses, as well as the bidirectional neuron–astrocyte signaling that mediates astrocyte–synapse interactions and their consequent effects on behavior, remain to be fully elucidated.

Herein, utilizing a reduced astrocyte–neuron coculture model system, our data indicate TrkB.T1-deficient astrocytes fail to form PAPs at synapses, suggesting that activation of the astrocyte TrkB.T1 receptor contributes to glutamatergic synapse recognition and subsequent PAP involvement. Analysis of cortical synaptosomes, which effectively capture both synapses and their surrounding PAPs, reveals a significant concentration of TrkB.T1 at the PAP. This enrichment is notably absent in mice lacking TrkB.T1, providing strong evidence for the localization of TrkB.T1 within the PAP. Furthermore, using a combination of protein and mRNA approaches as well as three-dimensional confocal microscopy, we demonstrate that loss of TrkB.T1 in astrocytes results in reduced glutamatergic synapse formation and astrocyte–synapse interactions in the motor and barrel cortex, and finally, conditional knock-out (cKO) of astrocyte TrkB.T1 alters motor learning and experience-dependent astrocyte–synapse interactions in these same regions. Altogether, our findings reveal astrocytes serve as a primary recipient of neuronally derived BDNF and provide novel insights into the role of astrocyte TrkB.T1 in modulating excitatory synapse formation, with important implications in neuronal development and plasticity. These insights underscore the necessity of considering the interplay between BDNF signaling, astrocyte function, and synaptic integrity, which may hold the key to understanding and addressing a range of neurodevelopmental and neuropsychiatric disorders.

## Materials and Methods

### Animals

All experiments were performed according to NIH guidelines and with the approval by the Institutional Animal Care and Use Committee at Virginia Polytechnic Institute and State University. All animals were maintained on a reverse 12 h light/dark cycle (lights on at 10 P.M., lights off at 10 A.M.). Food and water were available *ad libitum.* Wild-type (WT) *TrkB.T1^+/+^* and knock-out (KO) *TrkB.T1^−/−^* (Dorsey et al., 2006) C57/B6 mice were used for these experiments. These global TrkB.T1 mutant mice were generated by crossing a heterozygous *TrkB.T1^+/−^* male with a heterozygous *TrkB.T1^+/−^* female. Global TrkB.T1 mutants are referred to as global WT (TrkB.T1^+/+^) or global KO (TrkB.T1^−/−^) in the paper.

Astrocyte-specific cKO mice were generated in house. Aldh1l1-cre/ERT2 (Jackson Laboratory #029655) mice were crossed to TrkB.T1^fl/fl^ mice. To obtain experimental animals, homozygous TrkB.T1^fl/fl^ Cre^+^ males were crossed to homozygous TrkB.T1^fl/fl^ Cre^−^ females, or vice versa, a homozygous TrkB.T1^fl/fl^ cre^+^ female was crossed to a homozygous TrkB.T1^fl/fl^ cre^−^ male. This cross resulted in offspring that were all homozygous TrkB.T1^fl/fl^ and either Cre^+^ or Cre^−^ mice. TrkB.T1^fl/fl^ Cre^+^ and Cre^−^ mice were injected once daily with 4-OH tamoxifen between Postnatal Day (PND) 8–10, creating astrocyte-specific TrkB.T1 cKO mice and littermate, age-matched control mice. Tamoxifen was made to stock concentration of 20 mg/ml in 1:19 ethanol to canola oil via sonication for 60 min at 50°C. Injections were administered intraperitoneally at 20 µl prior to knowing the genotype of each animal. Astrocyte-specific conditional TrkB.T1 transgenic mice are referred to as conditional WT (control) and cKO (Aldh1l1-TrkB.T1 cKO) in the paper. *TrkB.T1^−/−^* and TrkB.T1^fl/fl^ mice were a generous gift from Dr. Lino Tessarollo (Dorsey et al., 2006).

### Primary neuron–astrocyte cocultures

Neurons were cultured from global WT (TrkB.T1^+/+^) PND 0–1 pups according to Holt et al. (2019a,b). Briefly, following cortical dissociation, the tissue was minced and enzymatically dissociated with a Papain Dissociation Kit (Worthington Biochemical) following manufacturer’s instructions. After obtaining a cell suspension of 150 µl, microglia and oligodendrocytes were first removed with 10 µl incubation with Cd11b^+^ (Miltenyi Biotec) and Mbp^+^ (Miltenyi Biotec) microbeads for 10 min at 4°C. The flow through was collected. Utilizing Miltenyi Biotec’s Neuron Isolation Kit, neuronal populations were isolated by incubating the cell suspension with 15 µl of biotinylated antibodies for 10 min at 4°C followed by a 10 min incubation with 15 µl anti-biotin microbeads. The cell suspension was then applied to a prepped LS column, and neurons were collected in the flow through of two washes. Neuronal cell number was determined, and 1.0–1.25 × 10^5^ cells were plated on 1 mm glass coverslips (Electron Microscopy Sciences; catalog #72290-03) in a 24-well plate (Falcon; catalog #353047). The coverslips were poly-l-lysine treated and laminin coated prior to plating. Neurons were maintained in neuronal maintenance media (Neurobasal media, 2 mM 1-Glutamax, and 1× B27). On the second day postplating, 2 µM cytosine arabinoside (AraC; Sigma-Aldrich; catalog #C6645) was added to reduce non-neuronal contamination. On the third day postplating, a media change was performed to remove AraC.

At 3 d in vitro (DIV), astrocytes from PND 3–5 global WT (TrkB.T1^+/+^) or global KO (TrkB.T1^−/−^) littermates were isolated and plated on top of the neurons at a density of 0.75–1.0 × 10^5^. Briefly, following cortical dissociation and after obtaining a cell suspension as described above, microglia and oligodendrocytes were first removed with 10 µl incubation with Cd11b^+^ and Mbp^+^ microbeads for 10 min at 4°C. The flow through was collected, and astrocytes were then acutely isolated using anti-ACSA-2^+^ (astrocyte cell surface antigen 2) Microbead Kit (Miltenyi Biotec). The flow through cell suspension was incubated in 15 µl of FcR blocking buffer for 10 min at 4°C followed by 15 µl of ACSA-2 microbeads for 10 min at 4°C. The cell suspension was then applied to a prepped LS column, and astrocytes were eluted from the column after two washes. Astrocyte cell number was determined, and astrocytes were then subsequently plated or added to the WT neuronal plates. Subsequent half media changes occurred every 3–4 d.

### Coculture experiments

#### Time points

Primary cocultures were fixed at DIV 3 and 10. Cells were washed with cold PBS followed by fixation with cold 4% paraformaldehyde (PFA) for 15 min at room temperature. Cells were washed with cold PBS three times before storing or initiating immunofluorescence.

### Primary coculture immunofluorescence

Cocultures were fixed at DIV 3 and 10. Following fixation, cells were incubated for 1 h in Fish Gel Blocking Buffer [2% Fish Gel (Sigma-Aldrich G741-100G), 0.2% Triton X-100 (Sigma-Aldrich T9284) in PBS] at room temperature. Coverslips were then incubated with primary antibodies in diluted blocking buffer (1:3 blocking buffer in PBS) overnight at 4°C. Cells were then washed for 5 min (five times) in cold PBS before being incubated with Alexa Fluor 488, 546, and 647 secondary antibodies. Secondary antibodies were made in PBS, and cells were incubated in secondary antibodies for 1.5 h at room temperature and in the dark. Cells were washed for 5 min (five times) in cold PBS before being incubated in DAPI (1:1,000) for 5 min before final wash in PBS. Coverslips were mounted with ProLong Gold Antifade Reagent (Life Technologies; catalog #P36930) on glass slides (Thermo Fisher Scientific; catalog #12-550-15).

Astrocyte morphology was visualized with the membrane-associated protein GLAST, and pre- and postsynaptic elements were visualized with VGluT1 and Homer, respectively. Prior to image acquisition, the experimenter was blinded to experimental conditions. Fluorescent images were acquired with a Nikon A1 confocal microscope. Primary antibodies used were as follows: rabbit GLAST (Abcam; catalog #ab416, 1:5,000), mouse Homer (Synaptic Systems; catalog #160-011; 1:500), and guinea pig VGluT1 (Millipore; catalog #AB5905, 1:1,000). Secondary antibodies used were as follows: Alexa Fluor 488 (Invitrogen; catalog #A11008; 1 : 500), Alexa Fluor 546 (Invitrogen; catalog #A10036; 1:500), and Alexa Fluor 647 (Invitrogen; catalog #A21450, 1:500).

### In vitro cell counting

The 20× stitched images of the entire coverslips were generated on a Keyence BZ-X810 fluorescent microscope. Images were subsequently loaded onto QuPath v0.5.1. After scaling the image, the automatic detection wizard was utilized to isolate individual cells utilizing default settings. Features including cell max caliper and circularity were extracted and graphed either in R v4.5.1 or GraphPad Prism.

### In vitro PAPs and synapse evaluation

A machine learning algorithm was used on Imaris x64 9.8.2. for evaluation of PAPs and synaptic associations. The Imaris *Surface Module tool* was employed to create a 3D and segmented rendition of GLAST^+^ astrocytes. The smoothing factor, indicated in Step 2 of the module, was applied during the surface creation. In the same module, a machine learning algorithm was used to classify each segment on the astrocyte. The astrocyte was segmented based on the average diameter of randomly selected PAPs, which were defined as astrocyte elements that were high in GLAST intensity relative to the rest of the membrane, round elements that colocalized with pre- or postsynaptic elements, or both. Due to the varying intensities and shapes that these elements form, blinded experimenters provided Imaris with numerous examples of PAP elements and then allowed the module to classify the entire astrocyte surface based on the parameters and examples fed into the program. This allowed for an unbiased classification of astrocyte segments. Once the astrocyte was segmented and reconstructed, a 3D distance-based approach was used to quantify PAP and synapse interactions. The *Spots Module* in Imaris was used to create spots for each synaptic element based on the average diameter of each synaptic puncta. Next, a distance-based approach was used to quantify pre- and postsynaptic elements within 0.500 µm of each other. This provided a quantification of colocalized synapses. Next, the colocalized puncta were isolated in Imaris, and using a distance-based approach classification within the *Spots Module*, colocalized spots were classified into those that were within 0.750 µm of a PAP structure and those that were not. Finally, we quantified the percentage of PAPs that contained a colocalized spots.

### In vivo cell counting

The 20× stitched images of the motor cortex were generated on a Keyence BZ-X810 fluorescent microscope. Images were subsequently loaded onto QuPath v0.5.1. After scaling the image, the automatic detection wizard was utilized to isolate individual cells utilizing default settings in each channel, and total cell count was recorded.

### In vivo astrocyte morphology and synapse immunofluorescence

Astrocytes in conditional WT (control) and cKO (Aldh1l1-TrkB.T1 cKO) were fluorescently labeled via AAV.Lck-GFP virus—pAAV.GfaABC1D.PI.Lck-GFP.SV40 (Addgene Viral Prep # 105598-AAV5). PND 0–1 pups were intraventricularly injected with 2–3 ul 2.3 × 10^8^ virus following hypothermia-induced anesthesia. The injection site was determined following Kim et al. (2014) and as described previously by our lab (Holt et al., 2019a) with equidistance between the bregma and lambda sutures, 1 mm lateral from the midline, and 3 mm depth. A 10 µl Hamilton syringe and 32 G needle was used. Animals were left with their mother and subsequently injected with tamoxifen once a day between PND 8 and 11 as described above. At the time of collection, animals were anesthetized with intraperitoneal injections of 100 mg/kg of ketamine/xylazine and intracardially perfused with PBS followed by 4% PFA for 10 min. Brains were postfixed overnight in 4% PFA before being transferred to a sucrose solution (30% sucrose in PBS). Brains were stored at 4°C in sucrose until they sunk to the bottom of their conical tube (1–2 d). After sinking, brains were removed from sucrose, washed with cold PBS, and immersed in isopentane (Sigma-Aldrich; 2-methylbutane; catalog #320404) for 5–10 s. A small metal ramekin was half-filled with isopentane and placed on dry ice, allowing the isopentane solution to cool down to −45°C to enable tissue snap-freezing. Brains were then stored at −80°C for later sectioning or sectioned immediately on a Leica Microtome (Leica SM2010 R Sliding Microtome). Sections were 100 µm thick and stored in antifreeze solution (30% glycerol, 30% ethylene glycol in 1× PBS) at −20°C.

### Immunofluorescence

The 100 µm coronal sections containing the motor and somatosensory whisker barrel cortex were selected as detailed in Franklin and Paxinos. Sections were washed for 3 min (five times) with cold PBS before being blocked for 1 h in fish Gel Blocking Buffer [2% Fish Gel (Sigma-Aldrich G741-100G), 0.2% Triton X-100 (Sigma-Aldrich T9284) in PBS] at room temperature. Slices were then incubated with primary antibodies made in diluted blocking buffer (1:3 blocking buffer in PBS) overnight at 4°C. Following primary incubation, slices were washed for 5 min (five times) in cold PBS before being incubated with Alexa Fluor 555/546 and 647 secondary antibodies. Secondary antibodies were made in 1× PBS at a 1:500 concentration. Sections were incubated in secondaries for 1.5 h at room temperature before being washed for 5 min (five times) in cold PBS and incubated in DAPI (1:1,000) for 5 min before final wash in PBS. Sections were finally mounted with ProLong Gold Antifade Reagent (Life Technologies; catalog #P36930) on glass slides (Thermo Fisher Scientific; catalog #12-550-15) and covered with cover glass (catalog #12-548-5E, Thermo Fisher Scientific).

Primary antibodies used were as follows: mouse NeuN (Millipore; catalog #mab377; 1:500), rabbit Sox9 (Abcam; catalog #ab185966; 1:500), mouse PSD 95 (Abcam; catalog #AB2723; 1:500), guinea pig VGluT1 (Millipore; catalog #AB5905; 1:1,000), and rabbit VGluT2 (Abcam; catalog #AB216463; 1:1,000). Secondary antibodies used were as follows: goat anti-guinea pig 546 (Invitrogen; catalog #A21435; 1:500), goat anti-rabbit 546 (Invitrogen; catalog #A11010; 1:500), and donkey anti-mouse 647 (Invitrogen; catalog #A31571; 1:500).

### In vivo synaptosome preparations

Synaptosome isolations were completed as described in Dunkley et al. (2008). PND 22–28 control and Aldh1l1-TrkB.T1 cKO mice were killed via CO_2_, and their brains were rapidly dissected, and cortices were dissected in ice-cold bubbled 1× Krebs buffer (1.26 M NaCl, 25 mM KCl. 250 mM NaHCO_3_, 12 mM NaH_2_PO_4_, 12 mM MgCl_2_, 25 mM CaCl_2_), pH 7.2. Cortices were added to 2 ml Dounce glass homogenizers (Wheaton; catalog #357422) on ice with 1.5 ml freshly made homogenizing buffer [2.5 ml 4× gradient buffer (containing 109.54 g sucrose, 606 mg Tris, 5 ml 0.2 M EDTA, pH 7.4), 7.5 ml ddH_2_O, and 50 mM dithiothreitol] and homogenized with 10–15 strokes. The resulting supernatant was transferred to a 2 ml centrifuge tube and centrifuged at 1,000 × *g* for 10 min at 4°C. A 100 µl of the supernatant was taken and diluted in 300 µl of 1× Krebs buffer with phosphatase (Sigma-Aldrich; catalog #P5726) and protease (Sigma-Aldrich; catalog #P8340) inhibitors; this was stored as the whole cortex fraction. The remaining supernatant was transferred to another 2 ml centrifuge tube and spun at 16,000 × *g* for 20 min at 4°C. The supernatant was kept as the cytosolic fraction. The remaining pellet was resuspended in 700 µl of homogenizing buffer using a pipette and laid on top of a preprepared percoll (Sigma-Aldrich; catalog #P1644-100 ml) gradient of 3, 10, 15, and 23% from top to bottom in gradient buffer + 50 mM dithiothreitol in a 4 ml ultracentrifuge tube (Beckman Coulter; catalog #349622). Tubes were placed in a chilled Beckman TLA 100.3 rotor and then placed in an Optima Max-XP ultracentrifuge (Beckman Coulter; catalog #393315) and spun at 31,000 × *g* for 6 min at 4°C. Following ultracentrifugation, four bands and one pellet were identified. The third and fourth bands from the top were collected using a glass Pasteur pipette as the synaptosome fraction. The resulting synaptosomes were transferred to a fresh 2 ml centrifuge tube and washed twice with 1× Krebs buffer at 15,000 × *g* for 15 min at 4°C or until a solid synaptosome pellet was visualized. Once the synaptosomes pelleted, the supernatant was discarded, and the pellet was resuspended in 100 µl of Krebs buffer plus inhibitors. All fractions protein content was assessed through a Pierce BCA assay, diluted to 2 µg/µl in Krebs buffer plus inhibitors if needed, and stored at −80° until used for Western blotting.

### Western blot evaluation

Sample lysates were diluted to a concentration of 0.33 µg/µl with Krebs buffer and mixed with 3.75 µl of 4× Laemmli sample buffer (Bio-Rad Laboratories; catalog #161-0747) containing 200 mM dithiothreitol. Samples were incubated for 10 min at 65°C. Equal amounts of protein were loaded into each lane of a 4–20% gradient precast acrylamide SDS–PAGE gel (Bio-Rad Laboratories; catalog #4561096). Gels were loaded with 2 µl of Precision Plus Protein Standard Dual Color ladder (Bio-Rad Laboratories; catalog #161-0374), followed by 15 µl of Hek cell lysate for negative control and 15 µl of each sample. Proteins were separated at 200 V constant. Gels were transferred utilizing the Trans-Blot Turbo RTA nitrocellulose transfer kit (Bio-Rad Laboratories; catalog #170-4270), at 1.0 A constant for 30 min. Following transfer, membranes were blocked in blocking buffer [1:1 ratio of TBS (150 mM NaCl, 2.6 mM KCl, 24.7 mM Tris) and Intercept Blocking Buffer (LI-COR Biosciences; catalog #927-60001)]. Blots were incubated in primary antibody solution containing 1:1 ratio of TBST (TBS + 0.002% Tween 20) and Intercept Blocking Buffer overnight. The membranes were then rinsed three times for 5 min in TBST and then incubated with fluorescently conjugated secondary antibodies for 1 h. Blots were once again washed three times for 5 min and imaged under a LI-COR Odyssey Scanner 9120 Classic. Images were later analyzed under Image Studio, normalizing samples signal mean to area first then to in lane housekeeping genes and finally to the sham group. Primary antibodies used were rabbit Homer (1:5,000, Protein Tech; catalog #12433-1-AP), rabbit Kir4.1(1:5,000, Protein Tech; catalog #12503-1-AP), rabbit TrkB (1:5,000, Millipore; catalog #07-225), rabbit Glt1 (1:5,000, Millipore; catalog #AB1783), rabbit GRIN2B (1:5,000, Protein Tech 21920-1-AP), guinea pig Vglut1 (1:5,000, Millipore; catalog #AB5905), mouse MAP2 (1:2,500, Sigma-Aldrich; catalog #M1406), and mouse actin (1:10,000, Millipore; catalog #A5441). Secondaries utilized were goat anti-mouse 680 (1:10,000, LI-COR Biosciences; catalog #925-68070), donkey anti-guinea pig 680 (1:10,000, LI-COR Biosciences; catalog #926-68077), donkey anti-mouse 800 (1:10,000, LI-COR Biosciences; catalog #926-32212), and goat anti-rabbit 800 (1:10,000, LI-COR Biosciences; catalog #925-32211).

### RNA isolation and qPCR

Samples from our three developmental timepoints of interest were collected from conditional WT (control) and cKO (Aldh1l1-TrkB.T1 cKO). One PFA-fixed, 100-µm-thick coronal section was used for each animal (see above, in vivo astrocyte morphology and synapse immunofluorescence, for details on brain slicing). Two 750 µm tissue punches (one per hemisphere) containing the somatosensory cortex were collected. RNA was extracted using the manufacturer (Life Technologies)-recommended procedures for a RecoverALL Total Nucleic Acid Isolation Kit (catalog #AM1975) optimized for paraffin-embedded (FFPE) or PFA exposed samples. Following RNA isolation, 200 ng of RNA was reversed transcribed into cDNA using Bio-Rad Laboratories’ iScript SuperMix (catalog #1708841). All cDNA was normalized to 350 ng following conversion. The relative mRNA expression levels were determined using real-time quantitative PCR by Taqman Fast Advanced Master Mix for qPCR (catalog #4444557) and TaqMan specific probes. The ddCt method was used to quantify relative mRNA expression levels, with each normalization indicated where appropriate.

### In vivo imaging and analysis

#### Synaptic puncta in astrocyte regions

For imaging astrocyte PAP and synapse histology, *Z*-stack confocal images were acquired from the layer II/III motor cortex and layer IV whisker barrel cortex using a Nikon A1 confocal microscope with 60×/NA 1.4 oil immersion lens and at 5× digital zoom. eGFP-expressing astrocytes and the synaptic puncta within the astrocytes were obtained from both hemispheres. The astrocytes were sampled at a 2,048 × 2,048 frame size, 0.225 µM step size, and a 3 µM-total *Z*-stack was imaged and generated. Two to three images from each hemisphere and both hemispheres were imaged per animal for a total of three to four images per animal. Imaris (Bitplane, x64 9.8.2) was used to quantify synaptic punctate at a three-dimensional resolution according to the published literature (Fogarty et al., 2013; Kuljis et al., 2019; Simhal et al., 2019; Zhou et al., 2019; Haggerty et al., 2023). To examine synapses within an astrocytic region, eGFP^+^ astrocytes were located, and a 10 × 10 × 3 µm (100 µm^3^) region of interest (ROI) at least 10 µm from the cell body and closer to the distal boundaries of the astrocyte territory was randomly selected. Using the Imaris *Surface module*, a surface reconstruction of the eGFP^+^ astrocyte leaflets was built. For Spot Detection, we followed similar procedures as described before (Fogarty et al., 2013; Kuljis et al., 2019; Simhal et al., 2019; Zhou et al., 2019; Haggerty et al., 2023). Briefly, the presynaptic (VGluT1 and VGluT2) and postsynaptic (PSD95) punctate were detected using the Imaris *Spot Module* with 0.4 and 0.3 µm diameters, respectively, based on both the published literature and by manually finding the average diameter of our punctate across various samples. Only synaptic puncta/spots inside the astrocyte object were quantified. We determined how many presynaptic spots were directly opposed to postsynaptic spots with the distance of 0.750 µm. The spots module and distance-based classifications enabled Imaris to quantify the total number of presynaptic, postsynaptic, and colocalized puncta within our ROIs.

#### Astrocyte morphology and astrocyte synapse interactions

Layer II/III motor cortex and layer IV whisker barrel cortex astrocytes were imaged on a Nikon A1 confocal with 60× oil immersion lens and 2× digital zoom. Laser power and gain were adjusted for each astrocyte and corresponding synaptic puncta. eGFP-expressing astrocytes that were visually isolated from neighboring astrocytes were sampled at a 2,048 × 2,048 frame size, 1 µm *Z*-step size, and 2× digital zoom. Care was taken to ensure that the entirety of each astrocyte in the *Z*-plane was imaged. After acquisition, *Z*-stacks were 3D reconstructed and analyzed on Imaris. The *Surface Module* was utilized to create a surface reconstruction and to estimate astrocyte volume. Astrocyte–synapse interaction analysis was estimated as described (Testen et al., 2018; Testen et al., 2020; Kruyer et al., 2022; Walker et al., 2022). Briefly, the *Surface Module* in Imaris was used to generate a 3D reconstruction of each astrocyte, using the Lck-GFP channel signal to build the build the isolated surface. A special masked channel was generated using the 3D-surface rendering to isolate the Lck-GFP–expressing astrocyte from Lck-GFP background and other astrocytes. The masked channel was then utilized to perform colocalization analysis within Imaris. Colocalization between the masked Lck-GFP and the Alexa Fluor 546 or Alexa Fluor 647 signal, representing VGluT1/VGluT1 and PSD95 markers, respectively, was quantified. To remove the background of the synaptic signals, repeated measurements of unambiguous puncta of fluorescent signal intensity across multiple optical slices were obtained. The average of these measurements was measured to threshold the synaptic channels. Next, the masked Lck-GFP channel was selected as a ROI to focus on, and a new colocalization channel was generated which quantified the percentage of ROI (astrocyte eGFP signal) colocalized with the synaptic channel. This allowed for assessing the coregistry of fluorescent astrocyte and synapse signals within a given voxel and served as a proxy of astrocyte/leaflet proximity to pre- and postsynaptic elements.

### Accelerated rotarod

Motor coordination and learning were evaluated using the accelerating rotarod (Columbus Instruments). Randomly selected PND 30–35 mice were subjected to the accelerated rotarod for 3 consecutive testing days. During one testing session, mice were placed on the accelerated rotarod of 3.0 cm in diameter for four trials with a 2 min rest in between each trial. Each trial lasted for a maximum of 5 min, with the rod initially set to 5 rpm and a linear acceleration of 0.1 rpm/s. The amount of time for each mouse to fall from the rotating rod onto a foam pad 44.5 cm below the rod was recorded for each test trial. Latency-to-fall values from the four testing trials were averaged for each animal, and within-genotype session averages were calculated and reported.

### Statistical analysis

Statistical significance and figures were generated using GraphPad Prism 10.1.2. All data are represented as mean ± SEM, with *n*’s indicated as appropriate. The Shapiro–Wilk test was performed to determine the normality distribution of each dataset, and outliers were determined using GraphPad Prism's ROUT method with a *Q* = 5%. For data where only one comparison was needed, Student's *t* tests were performed. Two-way ANOVAs followed by Tukey's post hoc tests were performed for all data with multiple comparisons.

## Results

### Development of astrocyte–synapse interactions are mediated via TrkB.T1 receptor in vitro

**Figure 1.**
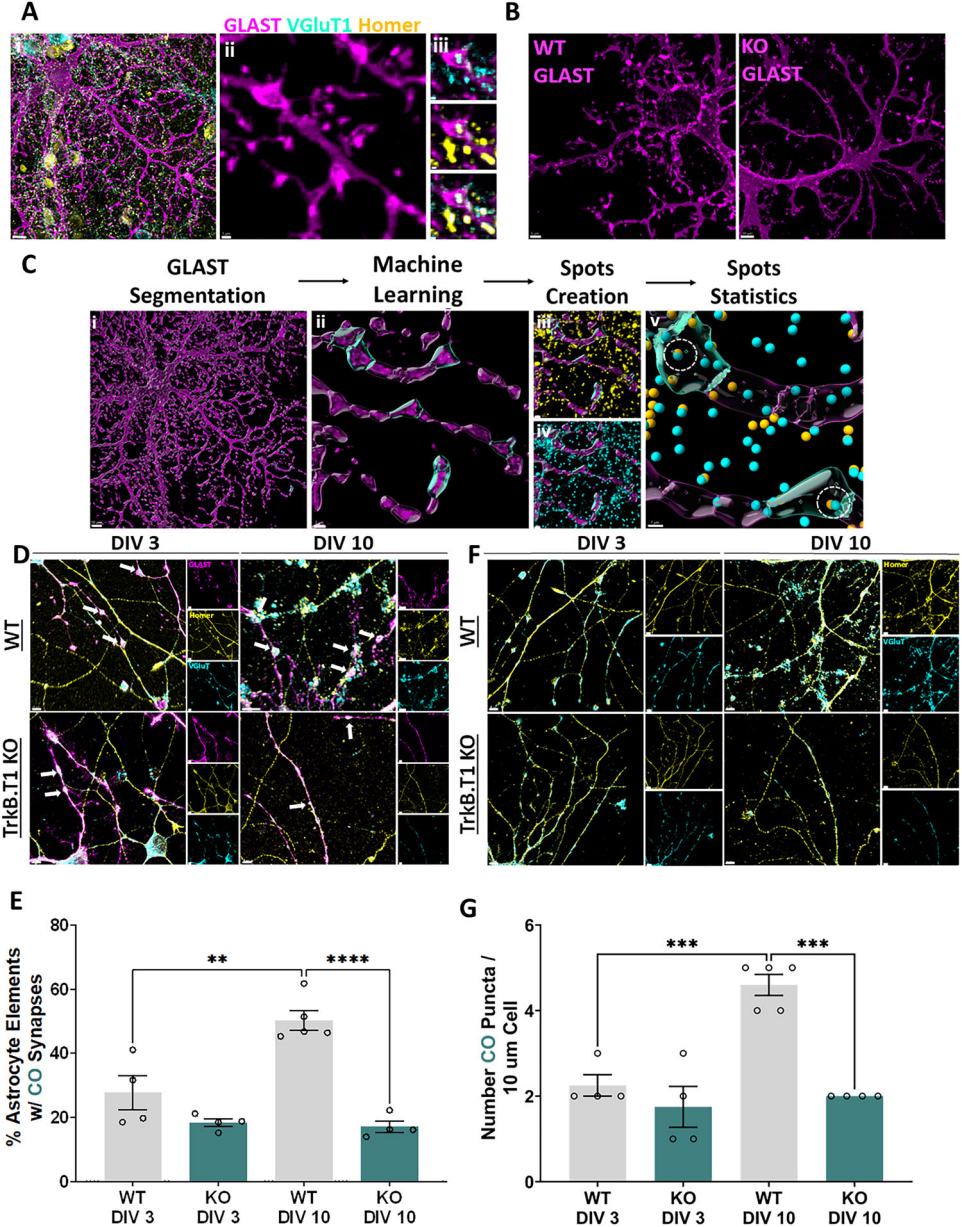
Astrocytes interact with developing neuronal synapses via TrkB.T1 in vitro. ***A***, ***i***, Representative image of GLAST^+^ astrocyte cocultured with neurons. Magnified astrocyte branches depicting high-intensity and/or cradle-like structures, termed PAP elements. These PAP elements were found to be associated with colocalized VGluT1 and Homer puncta. Scale bar, 10 μm (***i***), 1 μm (***ii***), 0.5 μm (Barbas et al., 2007). ***B***, Representative images of GLAST + TrkB.T1^+/+^ WT and TrkB.T1^−/−^ KO astrocyte. TrkB.T1^−/−^ KO astrocytes appeared to have a decrease in PAP elements. Scale bar, 10 μm. ***C***, Representative images of Imaris workflow to quantify astrocyte–synapse interactions in vitro. (***i***–***ii***) First, GLAST^+^ astrocyte is masked and segmented using machine learning classification to both identify and quantify PAP elements. (***iv***–***iv***) Imaris Spots Creation enabled identification and quantification pre- (VGluT^+^) and postsynaptic (Li et al., 2009) puncta. Scale bar, 2 μm (***v***). Analysis involved quantifying the percentage of PAP elements that contained a synapse, defined as distance-based colocalization of VGluT and Homer puncta. White dashed circles indicate a PAP associated with a synapse. Scale bar, 15 μm (***i***), 2 μm (***ii***–***iv***), 1 μm (***v***). ***D***, Representative images of TrkB.T1^+/+^ WT and TrkB.T1^−/−^ KO astrocytes cocultured with TrkB.T1^+/+^ WT neurons. Images depict the maturation of GLAST^+^ branches associated with neuronal processes between DIV 3 and DIV 10. Arrows represent PAPs. Scale bar, 4 μm. ***E***, Quantification of the percentage of PAPs that contained a synapse between DIV 3 and DIV 10. Mixed model ordinary two-way ANOVA + Tukey post hoc. ***F***, Representative images of synapses from TrkB.T1^+/+^ WT neurons in the presence of WT astrocytes or TrkB.T1^−/−^ KO astrocytes across development. Scale bar, 4 μm. ***G***, Quantification of synapse numbers between DIV 3 and DIV 10. Mixed model ordinary two-way ANOVA + Tukey post hoc. Data represented as averages ± SEM. Each dot represents an individual culture coming from two pooled animals with *n* = 3–5 cultures and *n* = 5 cells per culture (***E***) and 15 ROIs per cell (***G***). *p < 0.05; **p < 0.001; ***p < 0.001.

Given the canonical role of BDNF and TrkB in synapse formation and plasticity, we sought to investigate the role of astrocyte TrkB.T1 in synapse formation in vitro. Previously, we demonstrated in an astrocyte–neuron coculture model system that TrkB.T1^−/−^ KO astrocytes fail to support the formation of structural or functional glutamatergic synapses in WT neurons, indicating a potential role of TrkB.T1 in the formation of synapses (Holt et al., 2019a). To directly investigate the underlying mechanisms by which astrocyte TrkB.T1 regulates synapse formation and maintenance and how disruption of this process may contribute to the synaptic deficits observed, we examined the formation of PAPs and their interactions with synapses using primary astrocyte–neuron cocultures from TrkB.T1^+/+^ WT and TrkB.T1^−/−^ KO mice. Cortical TrkB.T1^+/+^WT neurons were cultured from P0–1 mouse pups and allowed 3 d to recover. Subsequently, TrkB.T1^+/+^ WT or TrkB.T1^−/−^ KO astrocytes were plated on top of the TrkB.T1^+/+^ WT neuron cultures. After maintaining cocultures for either DIV 3 or 10 (Chithiramohan et al., 2022), cocultures were PFA-fixed and confocal images of excitatory synapses (VGluT1^+^/Homer^+^) and astrocytes (GLAST^+^) were acquired (Fig. 1*A*). Immunolabeling for the astrocyte-specific GLAST, which served as a membranous label as opposed to a cytoskeletal label for astrocytes, revealed interactions between astrocytes and neurons, with astrocytes overlapping neuronal branches and extending finer ramifications into synaptic sites. We additionally observed astrocytes formed high-intensity GLAST^+^ hotspots that appeared to colocalize and/or sometimes appeared to cradle presynaptic (VGluT1) and postsynaptic (Homer) (Li et al., 2009) puncta (Fig. 1*A*). Notably, these structures were significantly reduced in cultures containing TrkB.T1^−/−^ KO relative TrkB.T1^+/+^ WT cultures (Fig. 1*B*). Given the decrease in neuronal synapses observed when neurons are cultured with TrkB.T1^−/−^ KO astrocytes (Holt et al., 2019a), we aimed to examine the role of BDNF/TrkB.T1 astrocyte signaling in mediating synapse formation and astrocyte–synapse associations. To examine the temporal dynamics of synapse and PAP development in vitro and the necessity of TrkB.T1, we PFA-fixed cocultures at different timepoints. We employed a machine learning algorithm in Imaris to both identify and classify the in vitro PAPs; these were defined as spots characterized by high-intensity GLAST^+^ labeling, made of a “donut” shape, or both. We henceforth refer to these PAPs as “astrocyte elements.” Imaris classifications then allowed for the quantification of astrocyte elements containing a synapse, defined as spots of colocalized pre- and postsynaptic puncta, obtained using the Imaris *Spots Module* (Fig. 1*C*). We established that synapses peaked at DIV 10 and thus quantified the upregulation of synapses between DIV 3 and 10. Two-way ANOVA revealed a significant effect of genotype (*F*_(1,13)_ = 42.54; *p*  <  0.0001; *N* = 4 DIV 3 WT; *N* = 4 DIV 3 KO; *N* = 5 DIV 10 WT; *N* = 4 DIV 10 KO, with *n* = 3–5 astrocytes per culture; Fig. 1*D*,*E*), as well as a significant interaction (*F*_(1,13)_ = 13.37; *p* = 0.0029) with development [(*F*_(1,13)_ = 10.70); *p* = 0.0061]. Post hoc Tukey’s multiple-comparison tests indicated that the percentage of astrocyte elements engaged with a synapse significantly increase between DIV 3 and 10 in TrkB.T1^+/+^ WT (*p* < 0.05) but not in TrkB.T1^−/−^ KO conditions (*p* = 0.1634). While there were no differences between TrkB.T1^+/+^ WT and TrkB.T1^−/−^ KO conditions at DIV 3 (*p* = 0.2462), at DIV 10, TrkB.T1^+/+^ WT cocultures had significantly more astrocyte–synapse interactions relative to TrkB.T1^−/−^ KO cocultures (*p* < 0.0001; Fig. 1*D*,*E*). As expected, the Imaris *Spot Module* tool analysis, which enables the quantification of synapses (Kuljis et al., 2019), revealed that the number of synapses also significantly increase between DIV 3 and 10 in TrkB.T1^+/+^ WT (*p* = 0.0003) but not in TrkB.T1^−/−^ KO conditions (*p* = 0.65). At DIV 10, there is a significant difference between TrkB.T1^+/+^ WT and TrkB.T1^−/−^ KO conditions (*p* = 0.0001; Fig. 1*F*,*G*). Altogether, we found a significant effect of genotype (*F*_(1,13)_ = 27.98; *p* < 0.0001; *N* = 4 DIV 3 WT; *N* = 4 DIV 3 KO; *N* = 5 DIV 10 WT; *N* = 4 DIV 10 KO, with *n* = 3–5 astrocytes per culture; Fig. 1*F*,*G*) as well as a significant interaction (*F*_(1,13)_ = 12.84; *p* = 0.003) with development (*F*_(1,13)_ = 19.68; *p* = 0.0007; Fig. 1*F*,*G*). We noted no differences in astrocyte or neuronal cell numbers based on Qupath analysis using DAPI labeling to assess nuclei number, circularity, or diameter between cultures containing WT or TrkB.T1 KO astrocytes (Supplemental Fig. 1*A*–*F*), suggesting differences in the cell number to do not account for our findings. These findings are in line with our previous publication in which we assessed astrocyte and neuronal cell numbers across the cortex from control and global TrkB.T1 KO mice and observed no differences (Holt et al., 2019a). Similarly, we also assessed total, neuronal, and astrocyte cell count with DAPI, NeuN, and Sox9, respectively, in control and Aldh1l1-TrkB.T1 cKO mice, finding no statistical differences in cell count (Supplemental Fig. 1*G*–*J*).

### TrkB.T1 is enriched at the synapse in vivo

To further elucidate the role of TrkB.T1 in astrocyte–synapse interactions and validate our in vitro observations, we next sought to investigate the subcellular localization of TrkB.T1 in vivo. Specifically, we aimed to determine whether TrkB.T1 is indeed concentrated at PAPs and synaptic interfaces in the brain, which would provide support for its proposed function in mediating astrocyte–synapse communication. Importantly, *Ntrk2*, which encodes for TrkB.T1, is highly and uniformly expressed in astrocytes across all brain regions as compared with other genes such as *Gfap* [Supplementary Fig. 2*A*,*B*; data mined from Allen Brain Cell Atlas (RRID:SCR_024440) https://portal.brain-map.org/atlases-and-data/bkp/abc-atlas].

**Figure 2.**
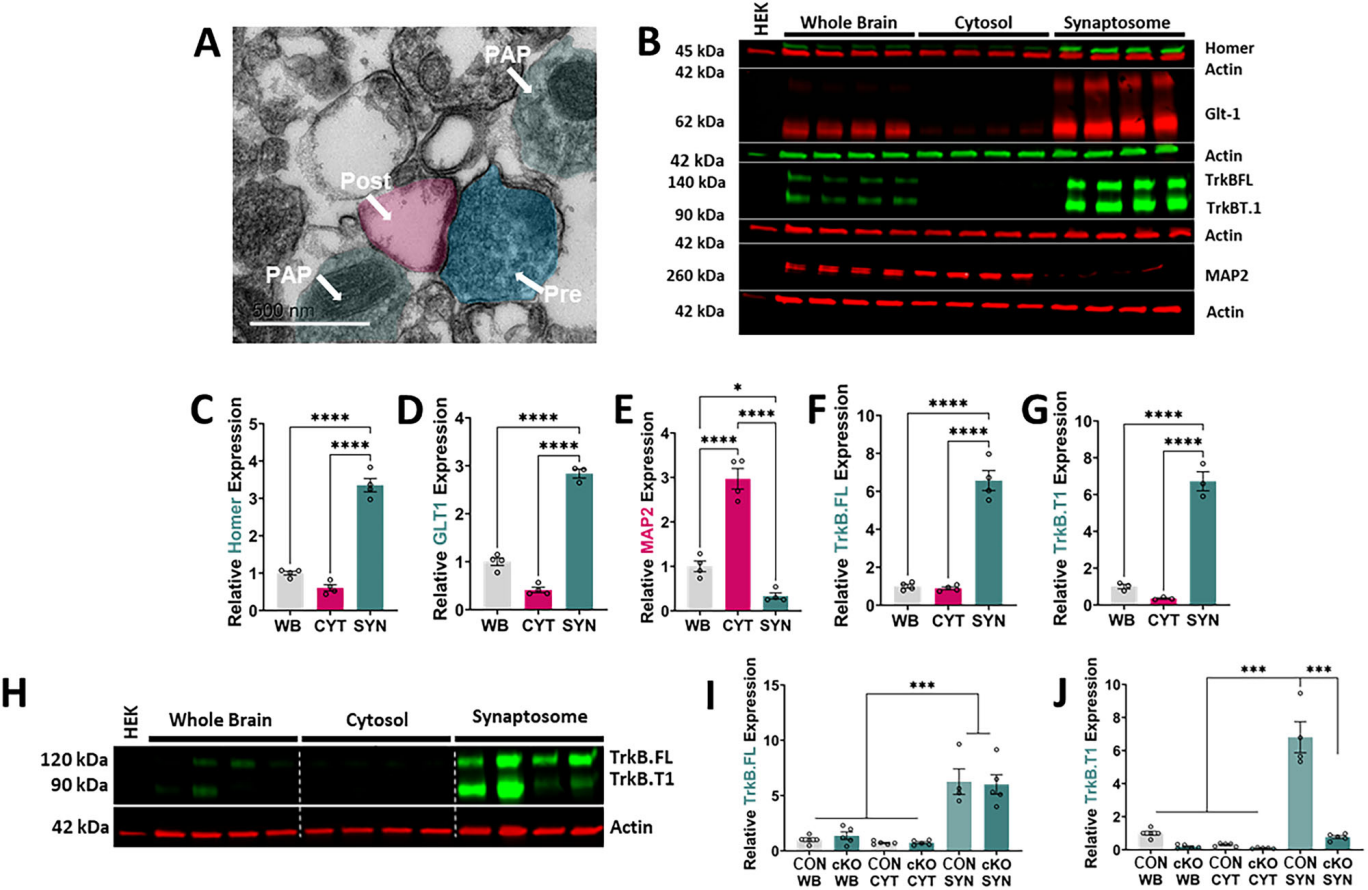
TrkB.T1 protein is enriched in PAPs in vivo. ***A***, Representative image of transmitted electron micrograph of sample synaptosome preparation. The TEM illustrates intact pre- and postsynaptic elements and PAPs (Landeiro et al., 2017). Scale bar, 500 nm. ***B***, Quality control Western blots for whole brain (WB), cytosol (CYT), and synaptosome (SYN) fractions and associated quantification in control tissue. A 5 µg of protein per lane. ***C–G***, Quantification of protein in ***A***. One-way ANOVA with Tukey post hoc. ***H***, Representative Western blots for WB, CYT, and SYN fractions of control and Aldh1l1-TrkB.T1 cKO animals. A 5 µg of protein per lane (***I,J***). Quantification of TrkB.FL and TrkB.T1 across various lysates of control versus Aldh1l1-TrkB.T1 cKO samples. Blots indicate that both TrkB.FL (***I***) and TrkB.T1 (***J***) are highly enriched in synaptosome preparations. However, conditional deletion of TrkB.T1 in astrocytes significantly decreases TrkB.T1 expression at the synapse (***J***), indicating that this protein is enriched in PAPs. (*n* = 3–5 animals). *p < 0.05; ***p < 0.001; ****p < 0.0001.

To achieve TrkB.T1 KO in astrocytes, homozygous TrkB.T1^fl/fl^-Aldhl1Cre^+^ or TrkB.T1^fl/fl^-Aldhl1Cre^−^ were injected with 20 µl (0.2 mg) 4-OH tamoxifen once daily (Zuo et al., 2018, Holt et al., 2019a) between PND 8–11. This protocol results in near complete elimination of TrkB.T1 gene and protein, with small effects of TrkB.FL at the gene, but not the protein level (Supplemental Fig. 2*C*–*K*). To specifically address the localization of TrkB.T1 at the synapse, we employed a synaptosome preparation. Synaptosome preparations are versatile tools for studying synapses; they contain intact pre- and postsynaptic membranes along with attached PAPs, enabling structural, functional, and biochemical analyses of the synaptic machinery and its components (Dunkley et al., 2008). Studies have demonstrated that these preparations collect PAP-associated proteins (Sapkota et al., 2022). Drawing from our earlier RNA sequencing findings (Holt et al., 2019a) and publicly available datasets (Zhang et al., 2014; Zhang et al., 2016; Chai et al., 2017; Kelley et al., 2018), we established that TrkB.T1 exhibits high expression in the mammalian cortex. Consequently, we anticipated that our synaptosome samples would be enriched in TrkB.T1. We validated our synaptosome preparations through electron microscopy and Western blotting for synaptic and cytosolic proteins (Fig. 2*A*,*B*). In the control WT samples, the postsynaptic protein Homer was enriched 4.43-fold more in the synaptosome (SYN) lysates than in the whole-brain (WB) lysates and 8.09-fold more strongly relative to the cytosol (CYT) lysates (Fig. 2*B*,*C*). The protein expression level of neuronal microtubule-associated protein-2 (MAP2) was 8.87-fold greater in CYT lysates than in SYN lysates and 2.96-fold greater than in WBs (Fig. 2*B*,*E*). In addition, we found high protein expression levels of astrocyte GLT1 (Fig. 2*D*), TrkB.FL (Fig. 2*F*), and TrkB.T1 (Fig. 2*G*), indicating that the synaptosome preparations effectively pulled down the tripartite synapse and its associated proteins, including TrkB.

When examining TrkB expression in Aldh1l1-TrkB.T1 animals, our results indicated that SYN lysates are enriched in TrkB.T1 expression (7.17-fold increase relative to WB lysate, *p* < 0.0001; Fig. 2*H*,*J*) in control animals. However, we observed 89% depletion of TrkB.T1 in SYN lysates of Aldh1l1-TrkB.T1 cKO animals relative to control animal SYN lysates (Fig. 2*J*), further confirming that astrocytes are the primary cell type expressing TrkB.T1. Importantly, no change in expression of presumably neuronal TrkB.FL was observed (Fig. 2*I*).These findings highlight the robust presence of the TrkB.T1 isoform at the tripartite synapse, particularly within PAPs, emphasizing its critical role in enabling astrocytes to respond to neuron-derived BDNF and facilitating astrocyte–synapse interactions.

### TrkB.T1 is implicated in the formation of glutamatergic synapses in the motor and barrel cortex

**Figure 3.**
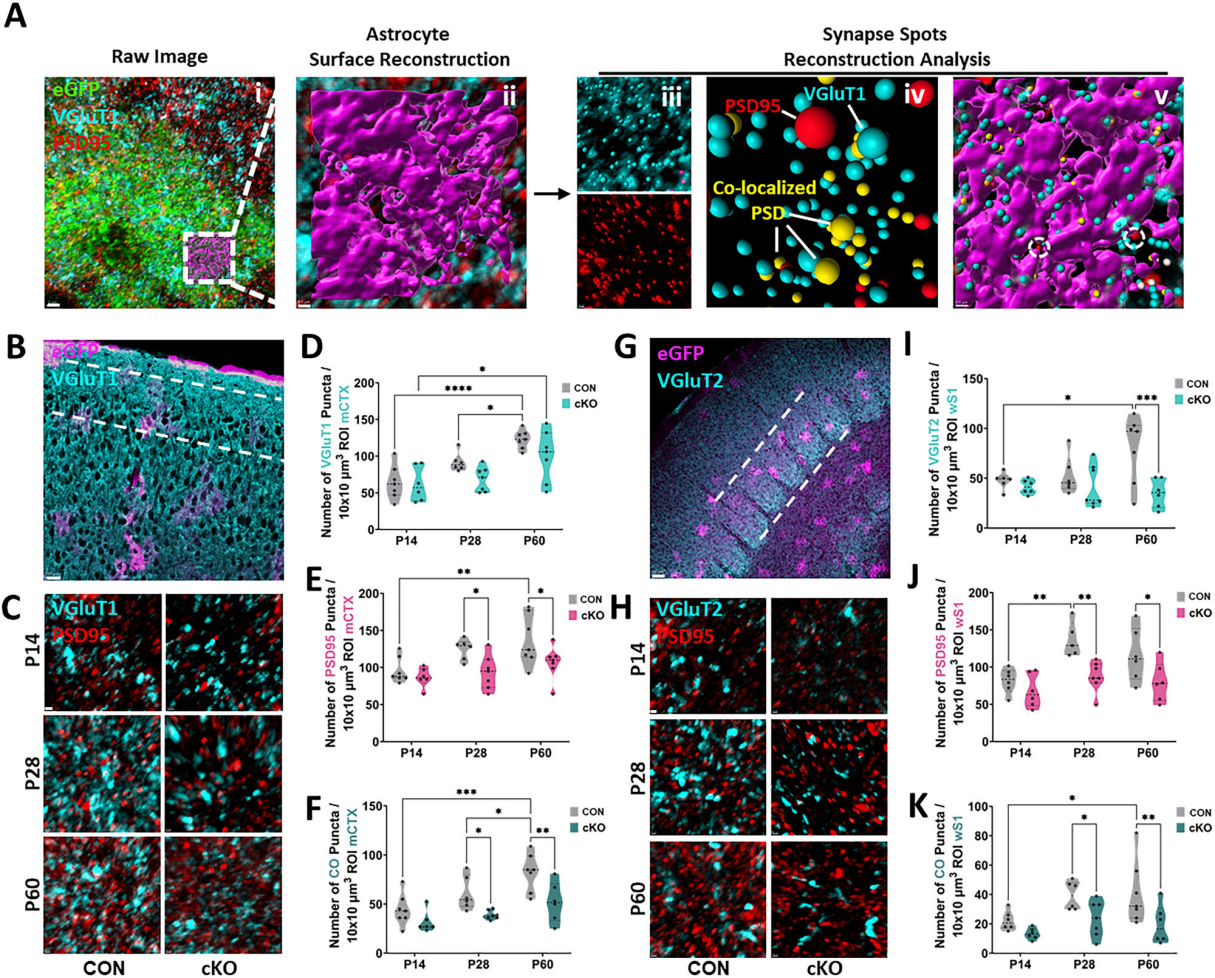
Conditional deletion of TrkB.T1 inhibits synapse formation in layer II/III of motor cortex and layer V barrels of barrel cortex. ***A***, Representative images of Imaris workflow to analyze pre- and postsynaptic elements. ***i***, Confocal stack of example lck-eGFP–expressing astrocyte and IHC for VGluT1 and PSD95 at 100×. Dashed square indicates (***ii***) selected ROI (10 × 10 μm^3^) within the astrocytic territory used to create a 3D rendering of the astrocyte's surface (Barbas et al., 2007). Raw synaptic fluorescent signal of VGluT1 and (***iv***) with 3D renderings of synaptic spots, depicted as blue spheres for individual VGluT1 puncta and red spheres for individual PSD95 puncta. Puncta creation parameters ranged from 0.3 to 0.5 μm estimated diameters. ***v***, Synaptic spots in the ROI. Cyan dots are VGluT puncta, red spheres are PSD95 puncta, and yellow spheres indicate PSD95 puncta colocalized and within 0.75 μm of a VGluT spot. High-throughput analysis allowed for the quantification of the total number of individual VGluT, PSD95, and colocalized puncta within ROIs. (scale bar: ***i***, 2 μm; ***ii***–***vi***, 0.5 μm). White circles indicate noncolocalized PSD-95 puncta. ***B***, Representative image of a coronal slice containing the motor cortex. Dashed lines indicate ROI. Scale bar, 30 μm. ***C***, Representative IHC images of VGluT1 and PSD95 of control and Aldh1l1-TrkB.T1 cKO animals across development. Scale bar, 0.5 μm (***D***) VGluT1 expression increases between PND 14 and 60 for both control and Aldh1l1-TrkB.T1 cKO animals. Two-way ANOVA with Tukey's post hoc. ***E***, Deletion of TrkB.T1 results in a reduction of PSD95 puncta at PND 28 and 60. A significant increase in the number of puncta occurs between PND 14 and 60 in control, but not Aldh1l1-TrkB.T1 cKO mice. Two-way ANOVA with Tukey's post hoc. ***F***, The number of glutamatergic synapses, colocalized VGlutT1/PSD95, increases across development in control animals. In Aldh1l1-TrkB.T1 cKO animals, there is a delay and only a trending increase in synapse numbers. At PND 28 and 60, there is a significant reduction in synapse numbers in Aldh1l1-TrkB.T1 cKO animals. Two-way ANOVA with Tukey's post hoc. ***G***, Representative image of coronal slice containing the barrel cortex. Dashed lines indicate ROI. Scale bar, 50 μm. ***H***, Representative IHC images of VGLuT2 and PSD95 of control and Aldh1l1-TrkB.T1 cKO animals across development. Scale bar, 0.5 μm. ***I***, VGluT2 expression increases between PND 14 and 60 in control animals and remains steady in Aldh1l1-TrkB.T1 cKO animals. By PND 60 the number of VGluT2 puncta are significantly lower in Aldh1l1-TrkB.T1 cKO animals. Two-way ANOVA with Tukey's post hoc. ***J***, PSD95 in the barrel cortex significantly increases in control animals between PND 14 and 28 and stabilizes and lowers by PND 60. Its expression remains steady in Aldh1l1-TrkB.T1 cKO animals across development, resulting in significant reductions by PND 28 and 60 when compared with control groups. Two-way ANOVA with Tukey's post hoc. ***K***, The number of glutamatergic synapses in layer IV of the barrel cortex significantly increases across development in control animals but remains unchanged in the Aldh1l1-TrkB.T1 cKO animals. This results in significant reductions in the number of synapses in Aldh1l1-TrkB.T1 cKO animals when compared with control. Two-way ANOVA with Tukey's post hoc. *N*  =  5–7 animals and *n* = 9–12 ROIs per animal (***D–F***, ***I–K***). *p < 0.05; **p < 0.001; ***p < 0.001.

Having established the localization of TrkB.T1 in PAPs, we next sought to elucidate how BDNF/TrkB.T1 signaling influences the development of synapses in vivo. We performed immunohistochemistry and confocal imaging of GFP-expressing astrocytes and probed for presynaptic vesicular glutamate transporters (VGluT1) and postsynaptic PSD-95 in layer II/III of the motor cortex (enriched in VGluT1-expressing corticocortical connections). We employed a membrane-tagged Lck-GFP expressed under the control of the GfaABC1d promoter, allowing for the visualization of astrocyte morphology and neuropil infiltration of PAPs (Morel et al., 2014, Holt et al., 2019a). Imaris analysis of astrocyte surface rendering and synaptic puncta detection allowed for a distance-based criterion for quantifying synapses (Fig. 3*A*; Kuljis et al., 2019). Here, a 10 by 10 µm^3^ ROI containing astrocyte signal was isolated and surface rendering was applied to the astrocyte membrane-tethered eGFP signal. Next, the Spots Module in Imaris was employed to isolate VGluT1^+^ puncta and PSD^+^ puncta, allowing for a distance-based approach to quantify puncta and synapses—or colocalized pre- and postsynaptic elements, encapsulated within 1 µm of the astrocyte membrane (Fig. 3*A*). We performed a mixed-effects two–way ANOVA to compare the effect of development and genotype on both the number of pre- and postsynaptic puncta and the number of colocalization events.

We imaged the motor cortex to examine if, at baseline, there are changes in synapse density in Aldh1l1-TrkB.T1 cKO mice. In layer II/III of the motor cortex (Fig. 3*B*), we observed a significant effect based on genotype for VGluT1^+^ puncta (*F*_(1,34)_ = 5.143; *p* < 0.05; Fig. 3*B–D*), PSD95^+^ puncta (*F*_(1,34)_ = 11.20; *p* < 0.005; Fig. 3*E*), and colocalized puncta (*F*_(1,33)_ = 18.48; *p* < 0.0005; Fig. 3*F*). We also observed a significant effect on development for all three synaptic components (VGluT1, *F*_(2,34)_ = 16.54; *p* < 0.0001; PSD95, *F*_(2,34)_ = 6.291; *p* < 0.005; CO, *F*_(2,32)_ = 11.33; *p* < 0.0002). Post hoc Tukey's multiple-comparison tests found significant developmental increases in VGluT1^+^ puncta for both control (PND 14–60, *p* < 0.0001; 95% C.I., −87.69, −29.46; PND 28–60, *p* < 0.05; 95% C.I., −1.576, 46.72) and to Aldh1l1-TrkB.T1 cKO animals (P14–60, *p* < 0.05; 95% C.I., −68.59, −7.981; PND 28–60, *p* < 0.05; 95% C.I., −60.83, −2.598). Importantly, the mean number of colocalized synaptic puncta was significantly higher in control animals at PND 28 (*p* < 0.05; 95% C.I., 4.292, 39.66) and PND 60 (*p* < 0.005; 95% C.I., 12.60, 47.97) when compared with Aldh1l1-TrkB.T1 cKO animals. As expected, the number of synapses in control animals significantly increased between PND 14 and 60 (*p* < 0.0005; 95% C.I., −37.88, 4.781) and between PND 28 and 60 (*p* < 0.05; 95% C.I., −42.78, −0.1233). In contrast, no significant increase in the number of colocalized puncta/synapses was observed across development in Aldh1l1-TrkB.T1 cKO animals.

To address if these changes were restricted to the motor cortex, we also evaluated the somatosensory cortex and focused on layer IV of the barrel cortex, enriched in VGluT2^+^ thalamocortical synapses. In the barrel cortex (Fig. 3*G*), we observed a significant effect based on genotype for VGluT2^+^ puncta (*F*_(1,32)_ = 9.688; *p* < 0.005; Fig. 3*G*–*I*) and PSD95^+^ puncta (*F*_(1,30)_ = 15.72; *p* < 0.0005; Fig. 3*J*). We observed a significant effect in the number of colocalized synapses in the barrel cortex, across genotypes (*F*_(1,32)_ = 13.28; *p* < 0.0005; Fig. 3*K*) and development (*F*_(2,32)_ = 4.274; *p* < 0.05; Fig. 3*K*). For convenience, enlarged images of synapses can be visualized in Supplementary Figure 3. Post hoc Tukey’s multiple-comparison tests found that the mean number of VGluT2^+^ puncta was significantly higher in control relative to Aldh1l1-TrkB.T1 cKO animals at PND 60 (*p* = 0.0004; 95% C.I., 21.74, 67.64), but not at other timepoints (Fig. 3*I*). No significant upregulation of VGluT2 or PSD95 puncta was observed for Aldh1l1-TrkB.T1 cKO animals, but a significant increase in VGluT2 and PSD95 was observed for control animals between PND 14 (early postnatal), 28 (juvenile), and 60 (early adult) timepoints. This indicates that in the barrel cortex, deletion of TrkB.T1 disrupts the developmental increase of pre- and postsynaptic elements, resulting in a significant decrease in colocalized puncta in juvenile and early adult Aldh1l1-TrkB.T1 cKO animals (PND 28, *p* = 0.0351; 95% C.I., 1.170, 30.35; PND 60, *p* = 0.0058; 95% C.I., 6.599, 35.78). Altogether, our data suggest that during early developmental critical time periods when astrocytes typically infiltrate the neuropil (Chung et al., 2015), removal of TrkB.T1 in astrocytes results in dysregulated formation of pre- and postsynaptic elements in the motor and barrel cortex. Consequently, this leads to a loss of synapses by PND 28 and 60.

### Astrocyte TrkB.T1 mediates astrocyte–synapse interactions across development

Our results indicate the important role of TrkB.T1 in the formation of synapses, yet, whether the lack of synapses in Aldh1l1-TrkB.T1 cKO animals is due to a change in astrocyte–synapse interactions is not known. As astrocytes mature morphologically, they extend leaflets to contact synapses, forming PAPs. These PAPs exhibit structural plasticity influenced by experience-dependent plasticity (Hirrlinger et al., 2004; Haber et al., 2006; Lavialle et al., 2011; Henneberger et al., 2020). While glutamate-induced activation of metabotropic glutamate receptors (mGluR1 and mGluR5) in PAPs triggers rapid structural changes (Lavialle et al., 2011), indicating glutamate is a driver triggering PAP plasticity, few studies have examined the molecular drivers of PAP–synapse interactions during development, with most focusing on experience-dependent plasticity or LTP models (Henneberger et al., 2020). Our previous work has established BDNF/TrkB.T1 signaling in astrocyte morphological complexity (Holt et al., 2019a). Thus, we next assessed the developmental effects on synapse associations with astrocytes. Here, the *Surface Module* in Imaris was used to generate a 3D reconstruction from the astrocyte Lck-GFP signal, and these reconstructions were used to create a masked channel isolating each astrocyte. Colocalization analysis was then performed between the masked Lck-GFP and VGluT1/2 or PSD95 voxels (Testen et al., 2020). This served as an evaluation of astrocyte–synapse associations.

**Figure 4.**
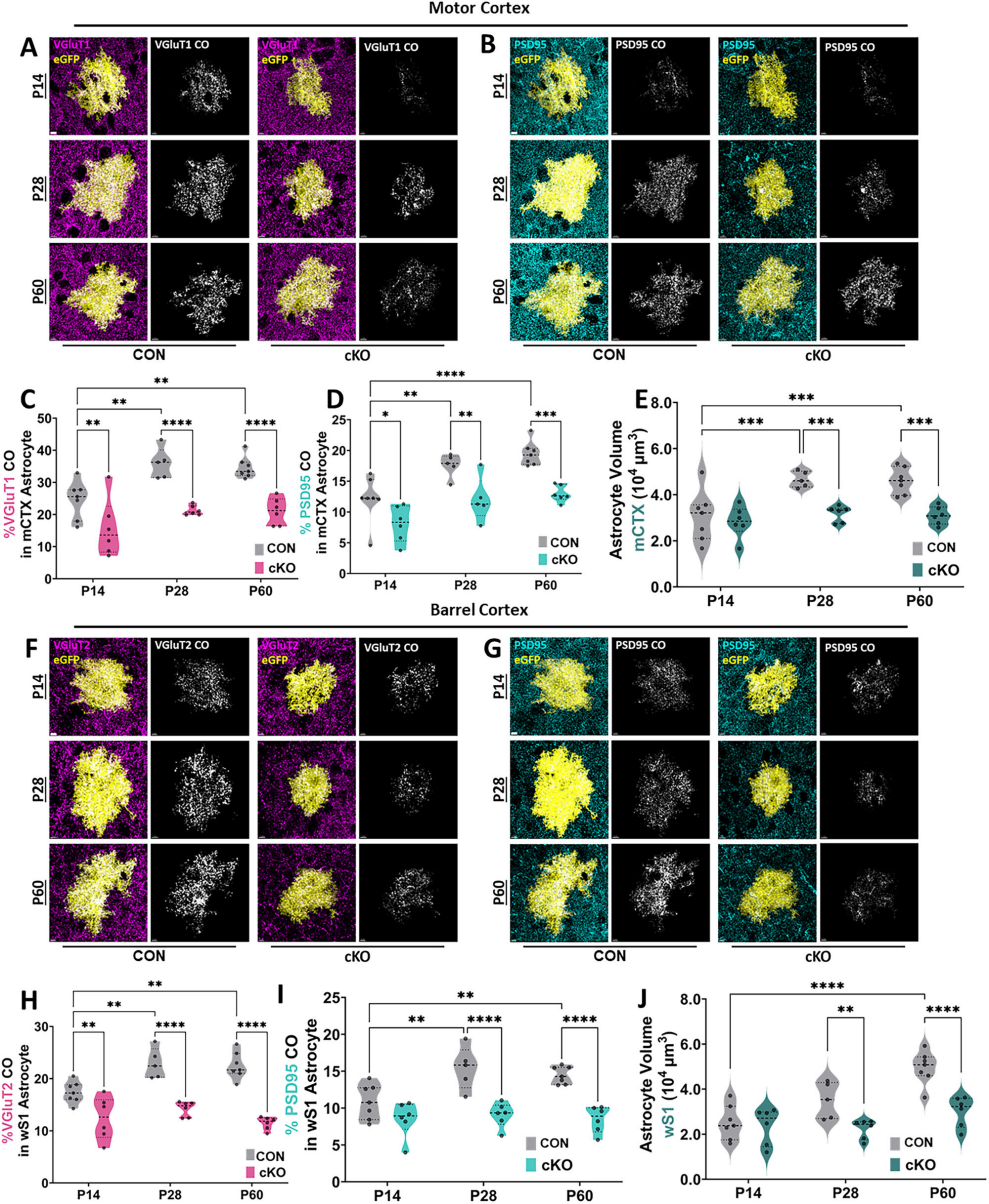
Conditional deletion of TrkB.T1 in astrocytes reduces astrocyte–synapse interactions. ***A***, Representative images of isolated Lck-GFP–expressing astrocyte with presynaptic marker VGluT1 and (***B***) postsynaptic marker PSD95 in the layer II/III motor cortex. When the two channels are merged, colocalized voxels (white) are quantified to determine the percentage of astrocyte membrane colocalized with (***A***) VGluT1 and (***B***) PSD95. Images depict control and Aldh1l1-TrkB.T1 cKO raw *Z*-series images and their corresponding colocalized voxels across development. Scale bar, 5 μm. ***C***, Astrocyte/VGluT1 contacts are significantly lower in Aldh1l1-TrkB.T1 cKO astrocytes across all stages in early development. The associations between PAP elements and VGluT1 increases in control animals but remain stable in Aldh1l1-TrkB.T1 cKO animals. ***D***, Astrocyte contacts with postsynaptic element PSD95 are significantly lower in Aldh1l1-TrkB.T1 cKO astrocytes across all stages in development. In control animals, astrocytes increase their associations with postsynaptic elements across developmental timepoints, but associations only trend toward an increase in Aldh1l1-TrkB.T1 cKO animals. ***E***, Overall morphometric properties of astrocytes increase across normal development but are lower in Aldh1l1-TrkB.T1 cKO animals. Two-way ANOVA with Tukey’s post hoc. ***F***, Representative images of isolated Lck-GFP–expressing astrocyte with presynaptic marker VGluT2 and (***G***) postsynaptic marker PSD95 in the layer IV barrel cortex. When the two channels are merged, colocalized voxels (white) are quantified to determine the percentage of astrocyte membrane colocalized with (***F***) VGluT2 and (***G***) PSD95. Images depict control and Aldh1l1-TrkB.T1 cKO raw *Z*-series images and their corresponding colocalized voxels across development. Scale bar, 5 μm. ***H***, PAP and VGluT2 interactions significantly increase across development in normal conditions but are consistently and significantly lower in Aldh1l1-TrkB.T1 cKO animals. ***I***, PAP and PSD95 interactions significantly increase across development in normal conditions but are consistently and significantly lower in Aldh1l1-TrkB.T1 cKO animals. ***J***, Overall astrocyte volumes significantly increase between PND 14 and 60 in control animals, with no increase between PND 14 and 28. Morphometric properties remain constant in Aldh1l1-TrkB.T1 cKO animals and are significantly lower across all time points when compared with controls. Two-way ANOVA with Tukey's post hoc. *N*  =  5–7 animals and *n*  =  3–4 astrocytes per animal (***C***–***E***; ***H***–***I***). *p < 0.05; **p < 0.001; ***p < 0.001.

In layer II/III of the motor cortex, a mixed model two-way ANOVA revealed a significant effect based on development and genotype for VGluT1 (development, *F*_(2,31)_ = 9.513; *p* < 0.005; genotype, *F*_(1,31)_ = 49.36; *p* < 0.0005; Fig. 4*A*–*C*) and PSD95 (development, *F*_(2,30)_ = 17.29; *p* < 0.0005; genotype, *F*_(1,30)_ = 33.55; *p* < 0.0001; Fig. 4*D*). We also measured astrocytic volumes, as a measure of astrocyte morphological complexity and maturation. We observed a significant effect based on development (*F*_(2,31)_ = 8.548; *p* < 0.005) and genotype (*F*_(1,31)_ = 24.62 = 24.62; *p* < 0.0005) on astrocyte volumes as well as an interaction (*F*_(2,31)_ = 3.883; *p* < 0.05; Fig. 4*E*). Across all developmental time points, multiple-comparison tests revealed a higher mean for the percentage of VGluT1 (PND 14, *p* < 0.005; 95% C.I., 2.846, 14.94; PND 28, *p* < 0.0005; 95% C.I., 8.069, 21.23; PND 60, *p* < 0.0005; 95% C.I., 7.582, 19.68) and PSD95 (PND 14, *p* < 0.05; 95% C.I., 0.93, 7.19; PND 28, *p* < 0.005; 95% C.I., 1.99, 9.10; PND 60, *p* < 0.0005; 95% C.I., 3.366, 9.626) associations in control astrocytes compared with Aldh1l1-cKO astrocytes. Overall astrocytic volumes were observed to significantly increase between PND 14 and 28 in control animals (*p* < 0.001), with no additional significant increases between PND 28 and 60 (*p* = 0.987) and an overall significant increase between PND 14 and 60 (*p* < 0.001).

In the barrel cortex, a mixed model two-way ANOVA revealed a significant effect based on development and genotype for VGluT2 as well as an interaction (development, *F*_(2,31)_ = 6.179; *p* < 0.005; genotype, *F*_(1,31)_ = 94.29; *p* < 0.0005; interaction, *F*_(2,31)_ = 4.573; *p* < 0.05; Fig. 4*F*,*H*). We observed a similar result for PSD95 (development, *F*_(2,31)_ = 4.787; *p* < 0.05; genotype, *F*_(2,31)_ = 50.24; *p* < 0.0005; interaction, *F*_(2,31)_ = 3.507; *p* < 0.05; Fig. 4*G*,*I*). We found a significant effect based on development and genotype and an interaction for astrocyte morphological maturation (development, *F*_(2,31)_ = 16.11; *p* < 0.0005; genotype, *F*_(1,31)_ = 22.75; *p* < 0.0005; interaction, *F*_(2,31)_ = 5.222; *p* < 0.05; Fig. 4*J*). Tukey's multiple-comparison tests revealed significantly higher VGluT2 localization in control astrocytes across all developmental timepoints (PND 14, *p* < 0.005; 95% C.I., 2.082, 7.893; PND 28, *p* < 0.0005; 95% C.I., 5.499, 11.82; PND 60, *p* < 0.0001; 95% C.I., 8.134, 13.95). Significantly higher levels of PSD95 and astrocyte associations in control animals were observed at later timepoints (PND 28, *p* < 0.0005; 95% C.I., 3.7772, 8.931; PND 60, *p* < 0.0001; C.I., 3.626, 8.366). Between PND 28 and 60, as we had observed previously in global TrkB.T1^−/−^ animals (Holt et al., 2019a), the mean astrocyte volume significantly increases in control animals (*p* < 0.005) and between PND 14 and 60 (*p* < 0.0001). Importantly, we did not observe any significant increases in barrel cortex astrocyte volumes in Aldh1l1-TrkB.T1 cKO animals.

Our data suggest that BDNF and TrkB.T1 signaling in development is an important signaling mechanism that mediates astrocyte–synapse interactions and morphological maturation. To address if these synaptic abnormalities were a result of reduced astrocyte engagement, we quantified the mRNA expression of common astrocyte-derived synaptogenic cues in the somatosensory cortex of control and Aldh1l1-TrkB.T1 cKO animals. Interestingly, we found reduced expression of the synapse-promoting cues *Thbs1* and *Sparcl1* and increased expression of the astrocyte-derived synaptic pruning factor *Megf10* at different timepoints in development (Supplemental Fig. 4*B*,*C*,*F*). We also observed significant reductions in PAP-related genes such as *Slc1a3*, Slc1a2, and *Kcnj10* (Supplemental Fig. 4*H*). We confirmed the change in gene expression corresponded to subsequent protein expression levels for GLT1 protein (Supplemental Fig. 4*I*,*J*). Using GLT1 immunohistochemistry, we identified reduced GLT1 overlap between AAV-GFP signal in Aldh1l1-TrkB.T1 cKO animals as compared with controls (Supplemental Fig. 4*I*,*J*). These results suggest that the loss of synapses may be due to reduced astrocyte interactions at the synapse, decreasing the ability for astrocytes to release or express synapse-promoting genes, or genes related to their ability to maintain ion and neurotransmitter homeostasis.

### cKO of astrocyte TrkB.T1 alters motor learning and experience-dependent astrocyte–synapse interactions

**Figure 5.**
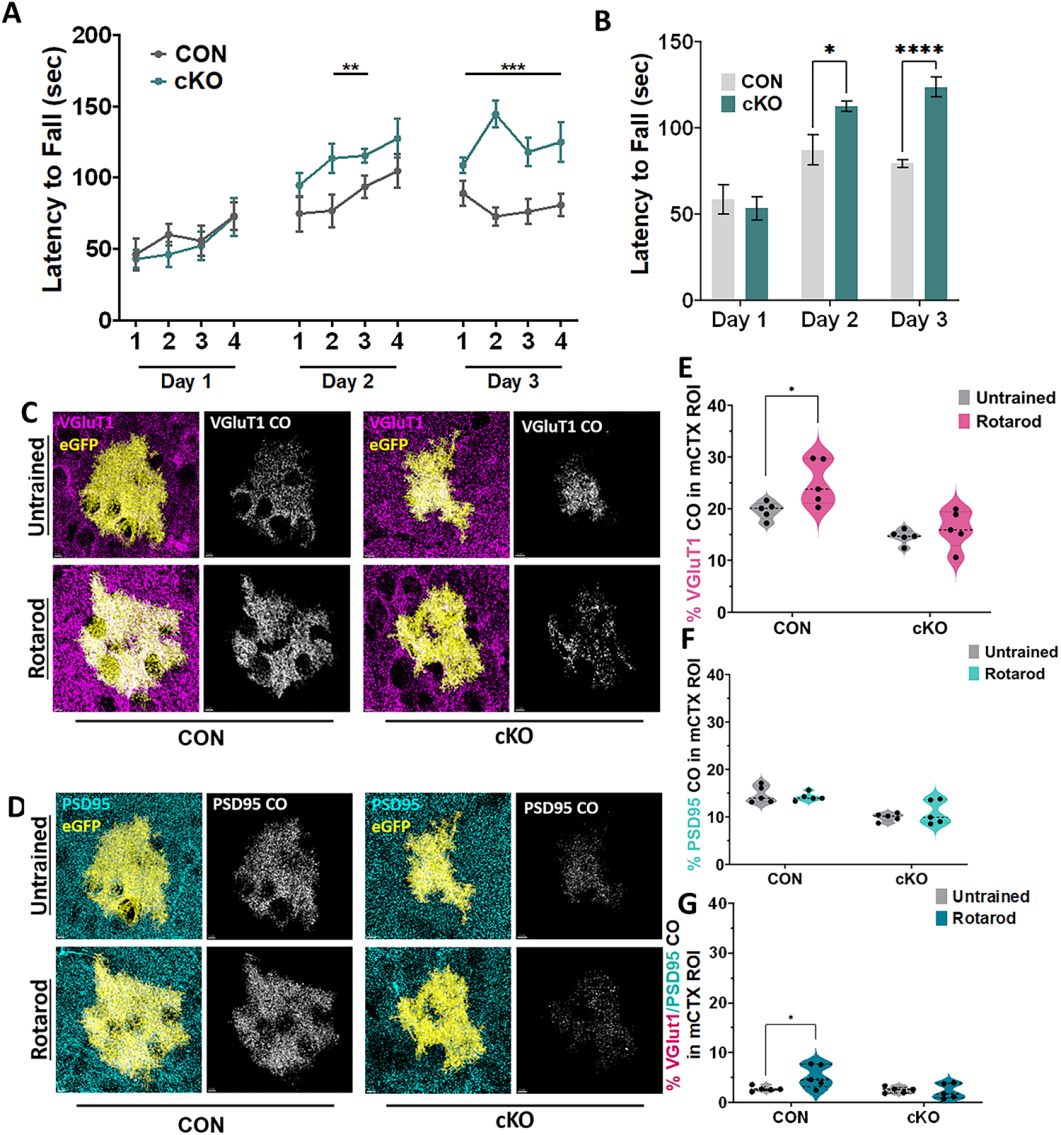
cKO of TrkB.T1 in astrocytes alters experience-dependent synaptic plasticity. ***A***, Latency to fall per trial across 3 d or averaged (***B***) per day. Aldh1l1-TrkB.T1 cKO mice exhibited significantly longer latency to fall. Two-way ANOVA with Tukey's post hoc. ***C***, Representative images of isolated Lck-eGFP–expressing astrocyte with presynaptic marker VGluT1 and (***D***) postsynaptic marker PSD95 in the layer II/III motor cortex. When the two channels are merged, colocalized voxels (white) are quantified to determine the percent of astrocyte membrane colocalized with (***E***) VGluT1, (***F***) PSD95, or (***G***) colocalized synapses. Images depict control and Aldh1l1-TrkB.T1 cKO untrained and trained raw *Z*-series images and their corresponding colocalized voxels across development. Scale bar, 5 μm. *T* test. *N* = 5 per condition and *n* = 3–4 astrocytes per animal. *p < 0.05; **p < 0.01; ***p < 0.001; ****p < 0.0001.

Considering the astrocyte synapse interactions observed in both the motor and somatosensory cortices across development, we next assessed whether loss of astrocyte TrkB.T1 would impact astrocyte structural plasticity/neuron interaction in response to an experience-dependent plasticity paradigm. TrkB.T1 has been implicated in motor function, where global KO of TrkB.T1 enhanced neuromuscular performance and nerve-evoked muscle tension (Kress et al., 2014). It is not known whether astrocyte-specific deletion of TrkB.T1 in astrocytes would similarly affect motor performance. We thus employed the rotarod performance test, a commonly used behavioral paradigm used to assess motor learning and performance. Here, mice were placed on an accelerating rotorod for four trials over a period of 3 d, with latency-to-fall values recorded for each animal for each trial. We identified, surprisingly, Aldh1l1-TrkB.T1 cKO performed significantly better on the rotarod test on Days 2 and 3 as compared with controls (Fig. 5*A*,*B*; Day 1, *p* < 0.9155; 95% CI, −17.17, 27,67; Day 2, *p* < 0.05; 95% CI, −47.74, −2.904; Day 3, *p* < 0.0001; 95% CI, −66.81, −21.98).

Motor learning tasks have been shown to increase synaptogenesis in the motor cortex (Kleim et al., 1996; Kleim et al., 2004). We thus queried whether the loss of astrocyte TrkB.T1 affects interactions between astrocytes and neighboring synapses in layer II/III as described above. Indeed, we identified that cortical astrocytes of control rotarod trained mice exhibited higher Vglut1 signal within astrocytes relative to untrained controls (*p* < 0.033079; Fig. 5*C*–*E*) but no difference in trained Aldh1l1-TrkB.T1 cKO mice versus untrained mice as analyzed by multiple unpaired *t* test analysis (*p* < 0.403848; Fig. 5*F*). No differences were observed between PSD-95 signal within astrocytes in either condition. However, the percentage of overlapped Vglut1 and PSD-95 signal within astrocytes was significantly higher in trained control mice versus untrained controls (*p* < 0.047749), but not in Aldh1l1-TrkB.T1 cKO mice (*p* < 0.751286; Fig. 5*G*). This suggests the role of astrocytic TrkB.T1 in experience-dependent synaptic plasticity. Similar experiments in vivo, using chemogenetic or other approaches to promote activity, would further strengthen the conclusion. Collectively, our findings from in vitro cocultures, synaptosome preparations, in vivo three-dimensional confocal microscopy and behavioral analyses converge to establish TrkB.T1 as a critical mediator of astrocyte–synapse interactions, demonstrating an enrichment in PAPs and its essential role in maintaining glutamatergic synapses and astrocyte–synapse interactions and plasticity in the cortex, thereby underscoring the fundamental importance of astrocytic BDNF/TrkB.T1 signaling in shaping synaptic landscapes in the mammalian brain.

## Discussion

In this study, we identify astrocyte-specific TrkB.T1 signaling as a critical regulator of astrocyte–synapse interactions and glutamatergic synapse development across cortical regions. Using complementary in vitro and in vivo approaches, we demonstrate that loss of astrocytic TrkB.T1 impairs PAP formation, reduces astrocyte engagement with synapses, and disrupts the developmental accumulation of excitatory synapses. In primary astrocyte–neuron cocultures, TrkB.T1-deficient astrocytes fail to increase astrocyte–synapse associations and appear to fail to support synapse maturation over time, despite normal neuronal and astrocytic cell numbers. In vivo, we identified TrkB.T1 is enriched at the tripartite synapse where it is positioned to respond to neuronally released BDNF, and astrocyte-specific deletion of TrkB.T1 leads to profound reductions in pre- and postsynaptic elements and their colocalization in both corticocortical and thalamocortical circuits. Together, these findings establish astrocytic BDNF/TrkB.T1 signaling as an essential, cell-autonomous mechanism linking astrocyte morphological maturation to the stabilization and maintenance of excitatory synapses during postnatal cortical development.

Astrocytes are essential for synapse development, promoting synaptogenesis through secreted cues and direct physical contact with synapses (Christopherson et al., 2005; Bae et al., 2011; Kucukdereli et al., 2011; Allen et al., 2012; Diniz et al., 2012). We previously showed that astrocytes predominantly express the truncated BDNF receptor TrkB.T1 that BDNF-TrkB.T1 signaling regulates astrocyte morphological maturation and that loss of TrkB.T1 impairs synapse formation in vitro (Holt et al., 2019a). Here, using a simplified neuron–astrocyte coculture system, we examined the temporal and spatial development of PAPs at excitatory synapses using GLAST-based membrane visualization. Temporally, we observed WT astrocytes envelop pre- and postsynaptic elements only when they demonstrate colocalization, while colocalized synapses with their associated PAPs increase over time in culture. In contrast, when WT neurons are cultured with TrkB.T1-deficient astrocytes, we identified marked reductions in colocalized synapse numbers and synapse PAP envelopment. These findings indicate that astrocytic TrkB.T1 signaling is required for effective astrocyte–synapse engagement and excitatory synapse maturation in vitro.

In vivo, conditional deletion of TrkB.T1 in astrocytes at the onset of synaptogenesis resulted in significant reductions in the development of glutamatergic synapses in the motor and barrel cortex and reduced astrocyte–synapse interactions. We observed significant reductions in synapse numbers and astrocyte–synapse interactions beginning at PND 28, coinciding with peak BDNF expression (Holt et al., 2019a; Esvald et al., 2023). Interestingly, in the ventromedial hypothalamus (VMH), BDNF depletion increased astrocytic process invasion and GLT-1 expression (Ameroso et al., 2022). This work suggests that in vivo, BDNF/TrkB.T1 signaling serves to mediate astrocyte–synapse interactions in regions such as the VMH, which undergoes plasticity in response to satiety-mediated BDNF expression levels (Ameroso et al., 2022). This contradiction may be due to depletion of BDNF rather than deletion of astrocytic TrkB.T1, where global depletion of BDNF will affect all TrkB isoforms, as opposed to just TrkB.T1. Furthermore, in a mouse model of temporal lobe epilepsy, modulation of astrocytic BDNF and TrkB significantly affects neuronal activity and cognitive function (Fernandez-Garcia et al., 2020), indicating an overall important role of astrocyte BDNF/TrkB in overall activity and behavior. Electrophysiological data have also indicated that neurons grown in vitro with TrkB.T1^−/−^ KO astrocytes have increased mEPSP amplitudes with lower frequencies, further indicating alterations in neuronal activity in response to TrkB.T1 deletion (Holt et al., 2019a). This may be a result of impaired astrocytic homeostatic capability, evidenced by a reduced propensity to interact with synapses (Fig. 4) and a reduction in proteins and genes critical to synaptic homeostasis, such as Glt-1 (Supplemental Fig. 4*I*,*J*). These experiments, however, should be repeated in future studies in vivo using the Aldh1l1-TrkB.T1 cKO mice. Furthermore, our results demonstrate differences in presynaptic changes between barrel cortices at P60 (and not earlier timepoints), but not in the motor cortex. This may manifest due to presynaptic marker choice, we chose VGluT2, as it labels the barrel cortex and, specifically, thalamocortical inputs, whereas VGluT1 marks broader excitatory presynaptic populations (Liguz-Lecznar and Skangiel-Kramska, 2007; Garcia-Marin et al., 2013) Thus, region-specific synaptic input may be differentially disrupted in Aldh1l1-TrkB.T1 cKO animals. Our previous in vitro work in combination with our developmental and behavioral studies suggests that during development and in the context of rotarod experience-dependent plasticity, TrkB.T1 drives astrocyte structural plasticity. This is evidenced by both a reduction astrocyte morphology across development and a reduced propensity to interact with synapses in the context of development- and experience-dependent motor learning. Indeed, TrkB.T1 mediates Ca^2+^ signaling in astrocytes in response to BDNF (Rose et al., 2003). Furthermore, TrkB.T1 in cultured astrocytes mediates astrocyte plasticity via Rho GDP dissociation Inhibitor 1, presumably via Ca^2+^ signaling-mediated actin dynamics (Ohira et al., 2005). Although our current work is limited in the temporal resolution required to observe immediate structural plasticity, it offers insights into the role of TrkB.T1 astrocyte–synapse engagement across development.

Our studies show loss of TrkB.T1 in cortical astrocytes renders them deficient in their BDNF response, consequently reducing their interactions at developing synapses. Furthermore, loss of TrkB.T1 may alter experience-dependent plasticity in the context of motor learning. To our surprise, rotarod trained Aldh1l1-TrkB.T1 cKO animals performed better than rotarod trained controls, despite a lack of changes in astrocyte structural plasticity evidence (Fig. 5*E*–*G*). While not evaluated here, it is possible that ratio of mature to immature synapses in Aldh1l1-TrkB.T1 cKO animals may be greater due to enhanced glutamate spillover from reduced astrocyte contact. Indeed, Badia-Soteras et al. determined that the retraction of the PAP enhances fear memory formation (Badia-Soteras et al., 2023). Furthermore, it is generally accepted that one function of TrkB.T1 is a dominant negative inhibitor of TrkB.FL (Li et al., 1998; Haapasalo et al., 2001), sequestering free BDNF. Hence, reductions in astrocyte TrkB.T1 may lead to increased levels of free BDNF driving heightened neural plasticity in this behavior paradigm. Ongoing work is aimed at evaluating neuronal plasticity in these mice. This impairment of TrkB.T1 may affect the expression of synapse-promoting cues, ion channels, and neurotransmitters critical for astrocytic support of synapses. We observed decreases in mRNA expression of GLT-1, GLAST, and Kir4.1, presumably due to reduced PAP formation, which may contribute to the reduction in synapse numbers observed. In addition, glutamate-induced activation of metabotropic receptors in PAPs triggers rapid structural changes in PAPs in the context of experience-dependent plasticity (Lavialle et al., 2011; Henneberger et al., 2020), suggesting glutamate is a driver of PAP plasticity. Herein, we propose that BDNF/TrkB.T1 signaling is akin to glutamate signaling in PAPs, contributing to synapse maturation. Overall, our results suggest that TrkB.T1 is a requisite PAP protein for astrocytes to engage with synapses in the context of development, with future work aimed at assessing the impact of TrkB.T1 deficiency in the context of experience-dependent plasticity.

It is important to acknowledge the limitations of our study, notably, the use of confocal microscopy rather than higher-resolution techniques like electron microscopy. While PAPs are nanoscale structures that are difficult to visualize using conventional light microscopy (Baldwin et al., 2023), confocal microscopy has been a valuable tool to obtain metrics such as astrocyte territory size (Endo et al., 2022), neuropil infiltration volumes (Bushong et al., 2002; Stogsdill et al., 2017), and territory volumes (Baldwin et al., 2021; Eaker and Baldwin, 2022). The methods used here have previously been used to study astrocyte–synapse interactions (Scofield et al., 2016; Jones et al., 2018; Kruyer et al., 2019; Siemsen et al., 2019; Testen et al., 2019). Future work, including live imaging, is necessary to distinguish whether the observed effects arise from impaired synapse formation, reduced survival or maturation of newly formed synapses, or increased synapse elimination. Concerning changes in experience-dependent plasticity, additional experiments utilizing alternative behavioral approaches would further assert the role astrocyte TrkB.T1 plays in supporting glutamatergic synaptogenesis. cKO of TrkB.T1 in adult astrocytes may help delineate the role of TrkB.T1 in synaptic development versus synaptic plasticity. Our current study lays a framework for assessing the role of astrocyte TrkB.T1 in neural plasticity during development and in the context of experience-dependent plasticity.

In conclusion, this study establishes a critical role of astrocytic TrkB.T1 signaling in the regulation of synapses and astrocyte–synapse associations during developmental time periods and adds to previous findings in the following ways: (1) investigations using neuron–astrocyte cocultures revealed impairments in the formation of PAPs and their interactions with excitatory synapses, (2) Aldh1l1-TrkB.T1 cKO astrocytes display reduced somatosensory and motor cortex synapse densities and astrocyte–synapse associations, and (3) Aldh1l1-TrkB.T1 cKO astrocytes showed enhanced motor learning but reduced synaptic plasticity. Our previous in vitro work in combination with our present data demonstrate that in the context of early development, astrocytes are a major recipient of neuronally derived BDNF, and loss of TrkB.T1 in astrocytes impacts astrocyte morphological maturation and astrocyte/neuron synaptic interactions and neuronal developmental plasticity. Ultimately, these studies lay the groundwork for elucidating the role of BDNF and astrocyte TrkB.T1 signaling and may unveil novel therapeutic avenues to target synaptic and behavioral abnormalities.
